# A Deep Unsupervised Learning Model for Artifact Correction of Pelvis Cone-Beam CT

**DOI:** 10.3389/fonc.2021.686875

**Published:** 2021-07-16

**Authors:** Guoya Dong, Chenglong Zhang, Xiaokun Liang, Lei Deng, Yulin Zhu, Xuanyu Zhu, Xuanru Zhou, Liming Song, Xiang Zhao, Yaoqin Xie

**Affiliations:** ^1^ State Key Laboratory of Reliability and Intelligence of Electrical Equipment, Hebei University of Technology, Tianjin, China; ^2^ Tianjin Key Laboratory of Bioelectromagnetic Technology and Intelligent Health, Hebei University of Technology, Tianjin, China; ^3^ Shenzhen Institutes of Advanced Technology, Chinese Academy of Sciences, Shenzhen, China; ^4^ School of Information Technology and Electrical Engineering, University of Queensland, Brisbane, QLD, Australia; ^5^ Department of Radiology, Tianjin Medical University General Hospital, Tianjin, China

**Keywords:** cone beam computed tomography, planning computed tomography, scatter correction, unsupervised deep learning, radiation oncology

## Abstract

**Purpose:**

In recent years, cone-beam computed tomography (CBCT) is increasingly used in adaptive radiation therapy (ART). However, compared with planning computed tomography (PCT), CBCT image has much more noise and imaging artifacts. Therefore, it is necessary to improve the image quality and HU accuracy of CBCT. In this study, we developed an unsupervised deep learning network (CycleGAN) model to calibrate CBCT images for the pelvis to extend potential clinical applications in CBCT-guided ART.

**Methods:**

To train CycleGAN to generate synthetic PCT (sPCT), we used CBCT and PCT images as inputs from 49 patients with unpaired data. Additional deformed PCT (dPCT) images attained as CBCT after deformable registration are utilized as the ground truth before evaluation. The trained uncorrected CBCT images are converted into sPCT images, and the obtained sPCT images have the characteristics of PCT images while keeping the anatomical structure of CBCT images unchanged. To demonstrate the effectiveness of the proposed CycleGAN, we use additional nine independent patients for testing.

**Results:**

We compared the sPCT with dPCT images as the ground truth. The average mean absolute error (MAE) of the whole image on testing data decreased from 49.96 ± 7.21HU to 14.6 ± 2.39HU, the average MAE of fat and muscle ROIs decreased from 60.23 ± 7.3HU to 16.94 ± 7.5HU, and from 53.16 ± 9.1HU to 13.03 ± 2.63HU respectively.

**Conclusion:**

We developed an unsupervised learning method to generate high-quality corrected CBCT images (sPCT). Through further evaluation and clinical implementation, it can replace CBCT in ART.

## Introduction

X-ray computed tomography (CT) is one of the most widely used medical imaging modalities because of its good image quality and high imaging speed. Unfortunately, radiation dose limits its application in clinics. In recent years, CBCT is widely integrated into modern linear accelerators for image-guided radiotherapy (IGRT). It can provide the latest anatomical information about patients, which is a useful tool to monitor the anatomical changes of patients during treatment (1–6). In addition, CBCT can facilitate adaptive radiation therapy (ART) by visualizing daily anatomic variations and making new radiotherapy plans for patients in real-time (7, 8). During radiotherapy, patients are usually affected by anatomical variations, such as tumor contraction or weight loss or inflammation (9, 10). These changes may lead to the difference between the planned dose and the actual dose, and increase the dose of organs at risk (OAR). Because of the large internal organ movement in pelvic cancer patients, IGRT cannot completely correct the positioning error. Therefore, the clinical implementation of ART is prospective to optimize pelvic cancer treatment.

As compared with planning CT (PCT), the image quality of CBCT is relatively poor, such as low signal-to-noise ratio, motion, and fringe artifacts (11, 12). The error of CT value between PCT and CBCT may be as large as 200 Hounsfield unit (HU) (13, 14). However, the acquisition of PCT may increase the treatment burden of patients, causing secondary radiation to patients. It will bring clinical benefits if we can directly use CBCT instead of PCT for ART.

To integrate CBCT into clinical ART scheme, the image quality of CBCT should be firstly improved. In recent years, many scattering correction techniques have been proposed and facilitate the application of CBCT in ART (15). These methods can be roughly divided into two categories, hardware correction and software correction. The methods of hardware correction aim to suppress the scattering in the process of projection data acquisition. Before the X-ray reaches the detector, the hardware, such as butterfly filter (16), anti-scattering grid (17), air gap (18), and other methods, is used to suppress the scattering, and the scattering correction is realized before the projection (19, 20). However, the quantum efficiency of the system is reduced. It often leads to the loss of important information and reduces the accuracy of reconstructed image. For software correction, deformable image registration (DIR) transforms PCT into CBCT to generate deformed plan CT (dPCT). Onozato et al. (21) and Veiga et al. (22) proposed a DIR method, which can calculate dose distribution similar to PCT scanning. However, in more complex anatomical changes, DIR may lead to incorrect results, as well as incorrect dPCT imaging. For example, the complex anatomical structure of lung (23) and pelvis (24, 25) and low soft-tissue contrast make it difficult to ensure the accuracy of dPCT dose. Monte Carlo (MC) simulation (26) is suitable for ART and is recognized as the gold standard for scattering correction. However, scanning an object to reach a detector requires simulating the true path of all the photons in the source of radiation, which is a time-consuming process and greatly reduces the clinical feasibility (27, 28). The analytical modeling method attempts to approximate the scattering distribution in the projection data by assuming that the scattering signal is the convolution function of the main signal and the scattering kernel (29). The method based on prior CT uses DIR to transform PCT into CBCT to obtain prior information for corresponding operation (30). The two histograms of CBCT image and PCT image are transformed into the same normalized uniform histogram. Then, the inverse equalization operation is carried out on the reference image (PCT) to correct the differences between them, such as density changes and energy stability, caused by scattering, organ movement, detector stability, and so on. By normalized matching correction of these artifacts, the density difference between CBCT images and PCT images is improved (31). All of the abovementioned methods require paired data sets of CBCT and PCT, which are difficult to obtain accurately. Dose calculation based on HU-D (HU-D) was feasible, and Richter (32) created a patient-specific HU-D table that allowed radiation dose calculation directly from the CBCT images without the need for PCT images. The proposed density assignment method first divides the image into different tissue categories (generally 2-6) and then assigns different densities to different tissue categories (33, 34). However, the method highly depends on the segmentation accuracy and image uniformity, which would affect the dose calculation.

Recently, deep learning has been widely used in the field of radiotherapy (35, 36). It can learn image features directly from data without parameter adjustment, which has strong robustness. Once the training of learning network is completed, the output results could be generated within a few seconds, which makes its application in adaptive radiotherapy desirable (37, 38). Because of these advantages, many studies are using deep learning networks to improve the quality of medical images and dose distribution. These methods are mainly divided into two categories: supervised learning and unsupervised learning.

For supervised learning, Maier et al. (39) used a U-net network to eliminate artifacts in CT and CBCT. Kida et al. (40) also applied U-net network to improve the uniformity of CBCT images and eliminate image artifacts. The root mean square difference (RMSD) in fat and muscle tissue decreased from 109 to 13 HU and from 57 to 11 HU, respectively. Hansen et al. (41) used an U-net network to correct the dose of CBCT. The 2% dose difference pass rate of VMAT is close to 100%, and the 2% dose difference pass rate of IMPT is between 15% and 81%. Similarly, Li et al. (42) introduced the residual blocks into the U-net network to improve the quality of CBCT, and the MAE between the synthetic CT (sCT) and CT decreased to about 20 HU. The above literature shows that all kinds of deep learning networks can effectively correct CBCT image artifacts, but these networks are supervised learning, and the data are required to be paired in the training process. However, it is difficult to obtain CBCT/PCT paired data, which limit the application of the supervised learning method.

In contrast, unsupervised learning can be trained using unpaired data. Wolterink et al. (43) first used CycleGAN to convert MRI of head and neck (H&N) to CT. Hiasa et al. (44) applied GAN to convert MRI to CT in pelvic and introduced gradient consistency loss to obtain a good conversion effect. Liang et al. (45) used CycleGAN network to synthesize CT images from CBCT, the average MAE of H&N patients was 29.85 ± 4.94 HU. Compared with other organs, such as the brain, H&N, the image synthesis of the pelvis is more challenging because of a variety of organs at risk (OARs) and the random movement of different organs. In this paper, we use the unsupervised learning network CycleGAN to directly convert CBCT into synthetic PCT (sPCT). The sPCT maintains the same anatomical structure as CBCT and has the HU accuracy of PCT. It should be emphasized that we do not require any corresponding relationship between CBCT and PCT. For instance, these images can be acquired from different patients on different dates. The only requirement is that they are scanned by the same CT scanning equipment.

## Materials and Methods

### Data Acquisition and Image Processing

In this study, we collected experimental data of 58 patients that were assembled as training (46) and testing (9) data sets from TCIA (47–49). Cone-beam computed tomography (CBCT) and planning CT (PCT) images were acquired from each patient, and the number of axial slices of CBCT and PCT per patient is approximately 88 and 130, respectively. After removing the data with extremely poor quality, 3,402 CBCT slices and 3,259 PCT slices were obtained. For CBCT, PCT images, a matrix size of 512 by 512 on the axial plane with a pixel size of 1 mm by 1 mm and a slice thickness of 1 mm. All images in the training data are normalized to the range of (−1, 1). During the training stage, because CycleGAN did not require paired CBCT and PCT images for training, there was no need for image registration between PCT and CBCT images, and CBCT and PCT slices were randomly shuffled in each period to eliminate the correspondence between different patients. During the testing stage, the results of our method should be evaluated using CBCT and PCT images ideally which are obtained at the same time to form a perfectly matched pair. Such a pair is almost impossible to obtain. In CBCT and PCT images, the anatomical structures were relatively similar. We used the Elastix toolbox (46) to perform deformed image registration and convert the PCT image to be aligned with the CBCT image. We used these deformed plan CT (dPCT) images as the ground truth to evaluate the sPCT generated from the CBCT images through the CycleGAN model.

### Overview of Cyclegan

**Figure 1:**
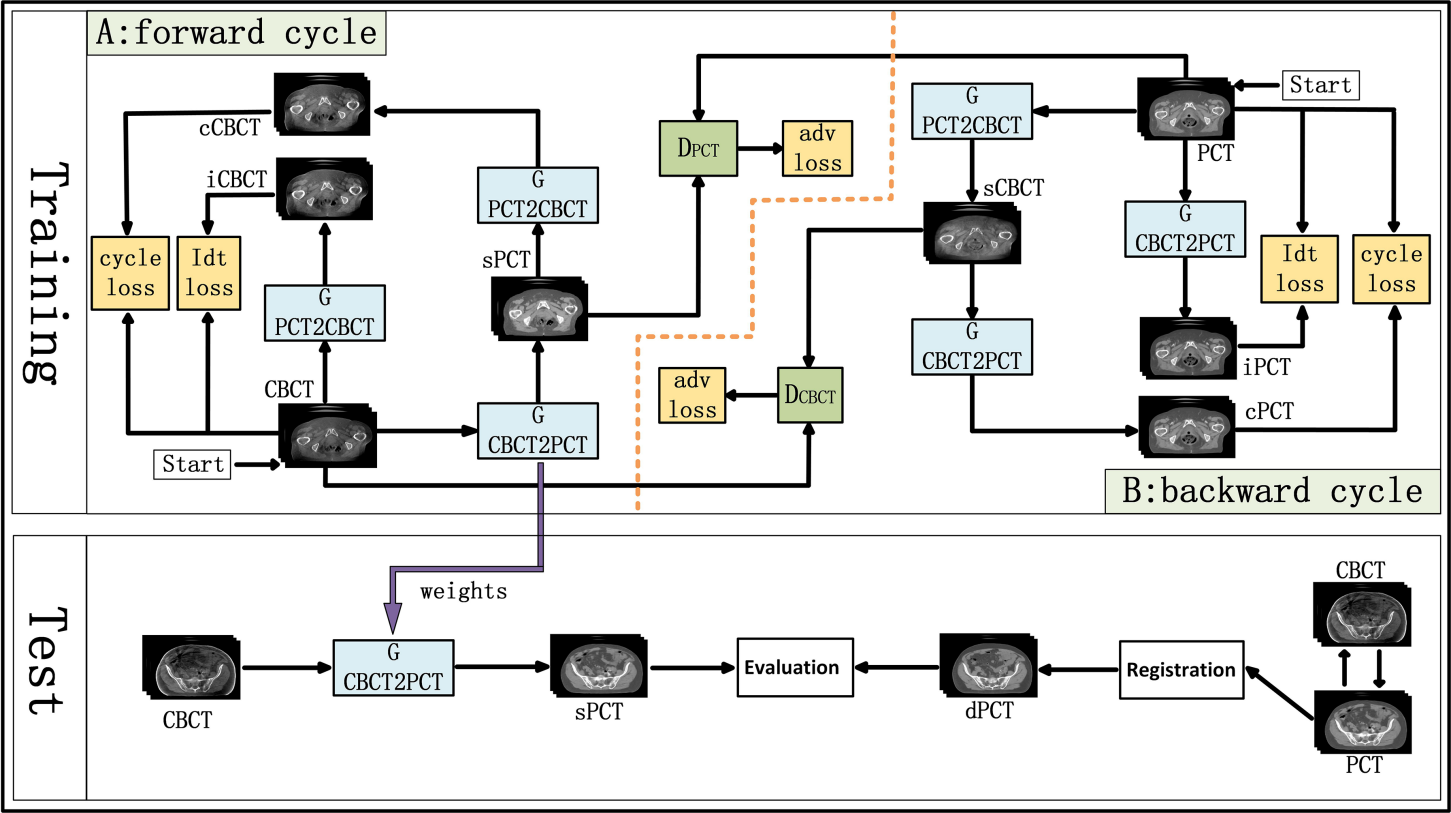
Schematic flow chart of the image synthesis using CycleGAN. The top area represents the training stage, and the bottom area represents the test stage. In the training phase, cone-beam computed tomography (CBCT) and plan CT (PCT) images are input into the network. The training network generates fake synthetic PCT (sPCT), cycle CBCT (cCBCT), identity CBCT (iCBCT) from CBCT, and fake synthetic CBCT (sCBCT), cycle PCT (cPCT), identity PCT (iPCT) from PCT. At the same time, the discriminator is trained to learn the relationship between sPCT and PCT, forcing the generator to produce sPCT closer to the PCT. SPCT is the generated fake image, the closer it is to dPCT, the better the quality of the generated image is; iCBCT is an image generated by CBCT input into *G_PCTtoCBCT_* generator to avoid the model migration of too many texture features; cCBCT is an image generated by sPCT input into G_PCTtoCBCT_ generator, with the purpose of one-to-one mapping of the model to ensure the consistency of the anatomical structure of sPCT and CBCT. In the test phase, CBCT images were input into the trained CycleGAN network to quickly generate sPCT images. The sPCT images are compared with the registered dPCT images.

CycleGAN is one of the major unsupervised learning methods. It consists of two parts, A and B, which form a forward cycle and a backward cycle, respectively. The forward cycle A indicates mapping from CBCT to PCT, and the backward cycle B refers to the reversed procedure. In addition, CycleGAN includes two generators (*G_CBCTtoPCT_* and *G_PCTtoCBCT_*) and two discriminators (*D_CBCT_* and *D_PCT_*. CycleGAN was trained to transform CBCT (input) into sPCT (output). **Figure 1** shows the architecture of the whole network. For part A, the training goal of the generator is to generate fake PCT (sPCT) images from CBCT images, which are as close to the real PCT as possible. And the training goal of the discriminator is to distinguish the sPCT images from the real PCT images. Ideally, *D_PCT_* would output sPCT images with marks of 0 and real PCT images with marks of 1. As mentioned above, the generator and discriminator in the network need to be constantly improved to make the sPCT images as close to the PCT images as possible, and the discriminator has a stronger identification ability, to achieve the balance of the game. The performance of generator and discriminator network is improved by using an adversarial loss function. To reduce the fluctuation in the training process, we refer to LSGAN (50) and use the least-squares loss function as the adversarial loss to make the whole training process more stable. The adversarial loss function is expressed as

(1) $$\begin{array}{l} \;{ℒ}_{adv}(G_{CBCTtoPCT},D_{PCT},CBCT,PCT)=\\ E_{PCT}[(D_{PCT}({\mathrm{PCT}}^{)}E1)2]+E_{CBCT}[D_{PCT}(G_{PCTtoCBCT}({\mathrm{CBCT}}^{)})2] \end{array}$$

(2) $${ℒ}_{\text{adv}}(G_{PCTtoCBCT},D_{CBCT},CBCT,PCT)=E_{\text{CBCT}}[{\left(D_{CBCT}(CBCT)-1\right)}^{2}]+E_{\text{PCT}}[D_{CBCT}{\left(G_{PCTtoCBCT}(PCT)\right)}^{2}]$$

where *G_CNCTtoPCT_* is the generator from CBCT to PCT, *D_PCT_* is the discriminator of sPCT and PCT. E is expectation, which is the most basic numerical feature to measure the concentration position or average level of the value of a random variable. E_CBCT_ is the expectation that the data belong to the real data CBCT, and E_PCT_ is the expectation that the data belong to the real data PCT. G aims to produce as realistic an image as possible to maximize the expectation value (over all E_CBCT_ and E_PCT_), whereas D tries to minimize the expectation value (over all E_CBCT_ and E_PCT_) by distinguishing real and synthetic image. The purpose of *G_CBCTtoPCT_* is to generate sPCT images as realistic as possible to maximize the value of *ℒ_adv_*(*G_CBCTtoPCT_*, *D_PCT_*, *CBCT*, *PCT*), *D_PCT_* constantly improves to distinguish PCT and sPCT images as much as possible to minimize *ℒ_adv_*(*G_CBCTtoPCT_*, *D_PCT_*, *CBCT*, *PCT*). In backward cycle B, PCT and CBCT are exchanged.

To further reduce the error, we couple the generating network (*G_CBCTtoPCT_*) with the inverse generating network (*G_PCTtoCBCT_*) and introduce the cycle consistency loss. *G_PCTtoCBCT_* could transform sPCT into cycle CBCT (cCBCT). By using L1 norm to constrain the distribution of cCBCT to be the same as the original CBCT. The difference between PCT and cCBCT is minimized, to minimize the difference between PCT and cycle CT (cPCT). In backward cycle B, PCT and CBCT are exchanged. The appearance of cycle consistency loss ensures the one-to-one mapping relationship. The cycle consistency loss function is expressed as

(3) $$\begin{array}{l} {ℒ}_{cyc}\left(G_{CBCTtoPCT},G_{PCTtoCBCT}\right)=E_{\text{PCT}}\left[\left||G_{PCTtoCBCT}\left(G_{CBCTtoPCT}\left(CBCT\right)\right)-\text{CBCT}|\right|\right]\\ \;+E_{\text{CBCT}}\left[\left||G_{CBCTtoPCT}\left(G_{PCTtoCBCT}\left(PCT\right)\right)-\text{PCT}|\right|\right] \end{array}$$

where the symbol ||*|| represents the L1-norm for CBCT or PCT.

To make CBCT and sPCT more similar in structure, identity mapping loss was added to the generator. The identity mapping loss function is expressed as

(4) $$\;{ℒ}_{identity}\left(G_{CBCTtoPCT},G_{PCTtoCBCT}\right)=E_{\text{PCT}}\left[\left||G_{CBCTtoPCT}\left(\text{PCT}\right)-PCT|\right|\right]+E_{\text{CBCT}}\left[\left||G_{PCTtoCBCT}\left(\text{CBCT}\right)-CBCT|\right|\right]$$

Therefore, by combining all the losses in the previous equations, the total loss of the network is

(5) $$\begin{array}{l} {ℒ}_{total}={ℒ}_{adv}\left(G_{CBCTtoPCT},D_{PCT},CBCT,PCT\right)\\ \;+\lambda_{cyc}\cdot {ℒ}_{cyc}\left(G_{CBCTtoPCT},G_{PCTtoCBCT}\right)+\lambda_{identity}\cdot {ℒ}_{identity}\left(G_{CBCTtoPCT},G_{PCTtoCBCT}\right) \end{array}$$

where *λ_cyc_*, *λ_identity_* is a regularization parameter, which controls the weight of cycle consistency loss and identity mapping loss respectively. The adversarial loss was used to map the distribution of the generated images to the distribution of the target domain images and facilitate the generated image closer to the real image. Cycle consistency loss prevents the two mappings from contradicting each other, which helps to improve the stability in the training process. Identity mapping loss prevents the generation of texture features with excessive image migration. In addition to training itself to generate the image, it also trains itself to distinguish the synthetic image from the real image. Finally, a good balance should be found among the three loss functions. In this study, we set *λ_cyc_* = 25 and *λ_identity_* = 0.5.

### Generators and Discriminators of CycleGAN

Generative Adversarial Networks (GAN) originally proposed by Ian Goodfellow (51), has two networks: generators and discriminators. The two networks are trained at the same time, the generator fools the discriminator by creating realistic images, and the discriminator is trained not to be fooled by the generator, and the two play against each other in a min-max game. Eventually, ideally, the generator can produce images that “look like the real image.”

**Figure 2:**
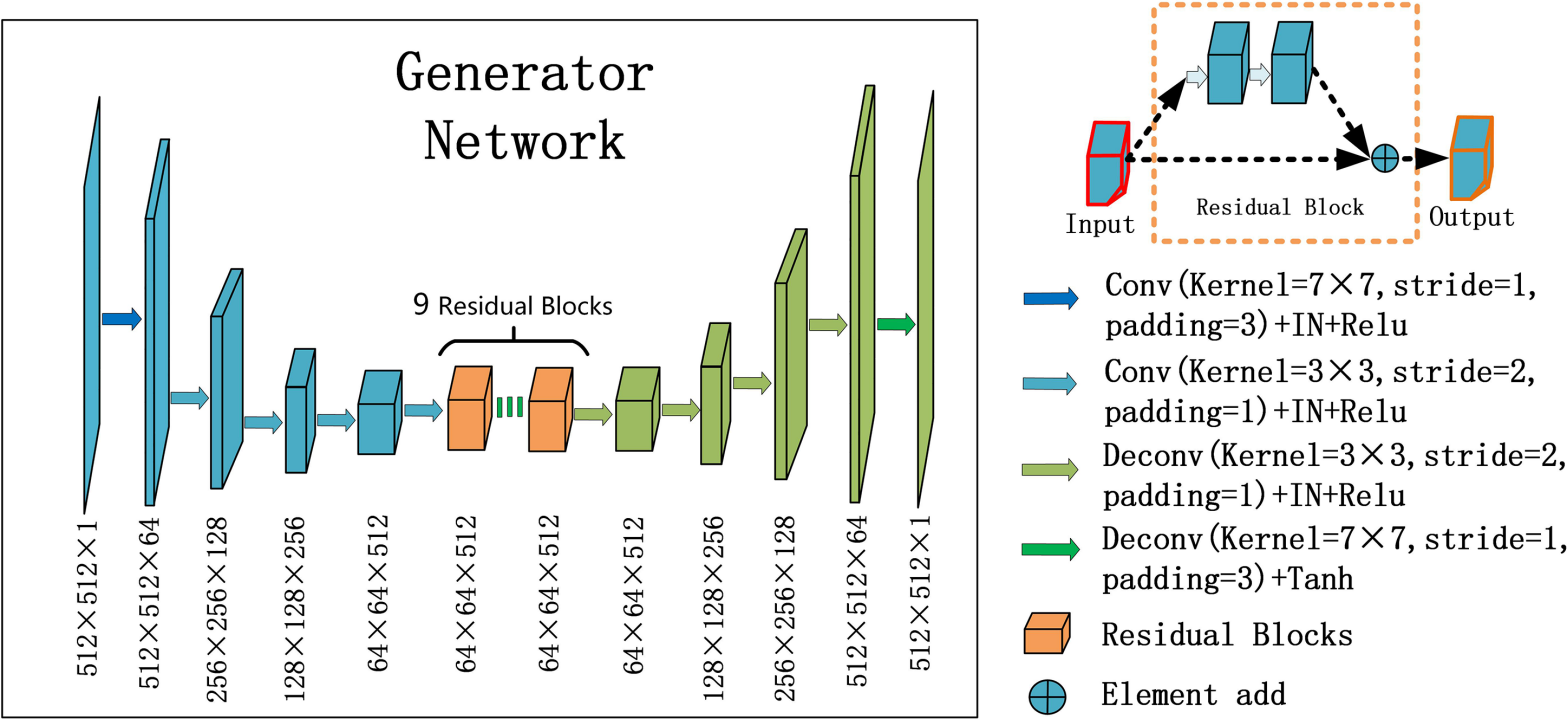
U-net and residual blocks are used for the generators in CycleGAN. The input data size is 512 ×512 ×1, and the output data size is 512 × 512 × 1. The first two digits represent the resolution, and the third digit represents the channel. Residual blocks are composed of 9 residual blocks.

For the generators *G_CBCTtoPCT_* and *G_PCTtoCBCT_* , we used an encoder-decoder network, which consists of three downsampling convolutional layers, nine residual blocks, and three up-sampling convolutional layers. After reducing the size of feature maps in three down-sampling convolutional layers, the feature maps passed through nine residual blocks, and then through three deconvolutional layers, and one Tanh activation layer as shown in **Figure 2**.

In each stage of the encoder, the width and height of the feature map are halved and the number of channels is doubled. The convolutional layers with stride 2 were used to replace the pooling layer. After the residual block is passed in the middle, the feature mapping of the encoder part is connected to that of the decoder part at the same stage, and the decoder part returns to the original image size by using the up-sampling convolutional layers.

Particularly, one convolution layer with a 7×7 kernel with stride 1 and 3 convolution layers with a 3×3 kernel with stride 1 were used for down-sampling to obtain 64×64 feature maps with 512 channels. And then, through nine residual blocks with a 3×3 kernel with stride 1, the size and channels of the feature maps remain unchanged. Then, 3 convolutional layers with a 3×3 kernel with stride 2 were used for up-sampling, and 512×512 feature maps with 64 channels were obtained. Finally, one convolutional layer with a 7×7 kernel with stride 1 was used to get 512×512 images. Except for the last layer, all convolution layers are activated with Relu activation function and normalized with instance normalization. The last layer was activated with Tanh activation function and without any normalization.

The residual block was composed of two convolutional layers (hidden layers), a residual connection and an element-wise sum operator. Among them, the hidden layer is to force learning the specific difference between CBCT and PCT, and the residual connection skips the hidden layer and directly adds elements to the results of the hidden layer.

**Figure 3:**
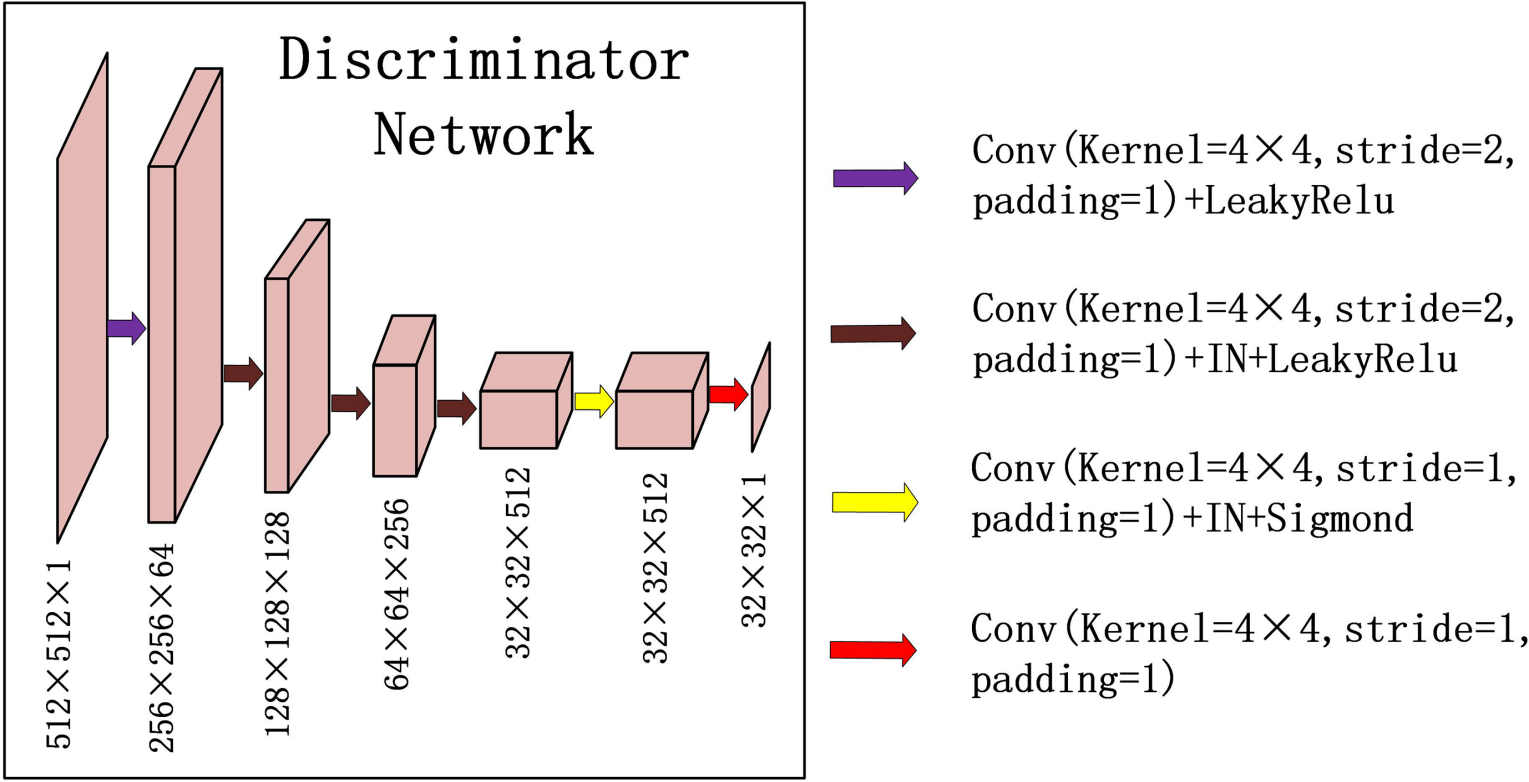
Down-sampling part of U-net is used for the discriminators in CycleGAN. The input data size is 512 × 512 × 1, and the output data size is 32 × 32 × 1. The first two digits represent the resolution, and the third digit represents the channel.

For discriminator *D_PCT_*(*D_CBCT_*), we used a typical down convolution network, as shown in **Figure 3**, *D_PCT_* was composed of five convolutional layers, the number of channels was reduced to 1, and the global average pooling was performed finally. The first four convolution layers were used to extract features, and the last convolution layer was used to judge whether the image is true or false. Specifically, in the process of downsampling, first, four convolutional layers with a 4×4 kernel with stride 2 were used to obtain 32×32 future maps with channels of 512. Then, after one convolutional layer with a 4×4 kernel with stride 1, the size and channels of the feature maps remain unchanged. After the last layer, we apply a convolutional layer to generate one-dimensional output.

Except that the first layer does not use instance normalization, all other layers use instance normalization and a LeakyRelu with a slope of 0.2 as the activation function.

### Technical Details

To improve the quality of the generated image, a morphological method (e.g., erosion, threshold, region growth, expansion, and filling operation combination) was used to set all pixels outside the pelvis contour of CBCT and PCT to 0. The model was trained and tested on NVIDIA TITAN XP GPU device with 12GB memory. All the networks were trained using the Adam optimization algorithm (52) with *β*_1_ = 0.5, *β*_2_ = 0.999 to stabilize training. The learning rate was set to 0.0002, and all weights are initialized from a random normal initializer with an average of 0 and a standard deviation of 0.02. In each optimization step, the slices of different patients were randomly selected. Instance normalization was employed with a batch size of 1. In the first 100 epochs, the learning rate remains unchanged, whereas in the next 100 epochs, the learning rate linearly drops to 0.

### Evaluation

In the CycleGAN model, the data are registered using the Elastix toolkit to generate the deformed PCT (dPCT), which is used as the ground truth for quantitative evaluation.

For quantitative comparison, sPCT is compared with dPCT using mean absolute error (MAE), root mean square error (RMSE), peak signal-to-noise ratio (PSNR), and structural similarity index (SSIM). The CT number accuracy, spatial uniformity, and structural similarity are evaluated for the generated image. MAE is used to calculate the average absolute error of all pixel values in two images, which reflects the difference between sPCT and dPCT. RMSE is used to measure the deviation between sPCT and dPCT. PSNR is used to evaluate image denoising. SSIM is a matrix of the structure information to compare the similarity of two images. The following are definitions of these matrixes between sPCT and dPCT:

(6) $$\text{MAE}=\frac{1}{M}\Sigma_{i,j}^{n_{sPCT}n_{dPCT}}|sPCT(i,j)-dPCT(i,j)|$$

(7) $$\text{RMSE}=\sqrt{\frac{1}{n_{sPCT^{n_{dPCT}}}}\Sigma_{i,j}^{n_{sPCT}n_{dPCT}}{\left(sPCT(i,j)-dPCT(i,j)\right)}^{2}}$$

(8) $$\text{PSNR}=10{\text{log}}_{10}\left(\frac{MAX^{2}}{\Sigma_{i,j}^{n_{sPCT}n_{dPCT}}(sPCT(i,j)-dPCT{\left(i,j)\right)}^{2}/n_{sPCT^{n_{dPCT}}}}\right)$$

(9) $$\text{SSIM}=\frac{\left(2\mu_{sPCT}\mu_{sPCT}+c_{1})(2\sigma_{sPCT,sPCT}+c_{2}\right)}{\left(\mu_{sPCT^{2}}+\mu_{dPCT^{2}}+c_{1})(\sigma_{sPCT^{2}}+\sigma_{dPCT^{2}}+c_{2}\right)}$$

where n_sPCT_, n_dPCT_ are the total number of pixels in sPCT and dPCT, respectively. MAX is the maximum intensity in sPCT and dPCT, *M* = *n_sPCT_*·*n_dPCT_*, *µ* is the mean value of the image, *σ* is the standard variation of the image, *C_1_* and *C_2_* are two variables that stabilize the division with a weak denominator.

## Results

### Qualitative Evaluation of Image Quality

**Figure 4:**
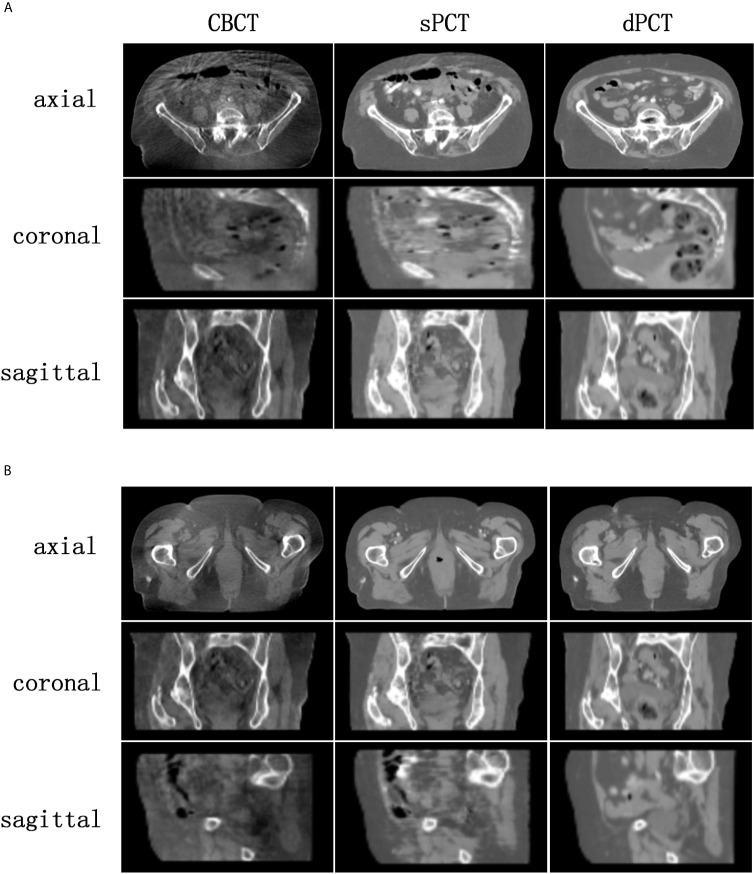
Comparison of image quality among CBCT, sPCT, and dPCT of two patients **(A, B)**. For each patient, the images in the top, middle, and bottom rows are axial, coronal, and sagittal, respectively. The left, middle, and right images represent CBCT, sPCT, and dPCT respectively. The display window is (700, 1300) HU.

**Figure 5:**
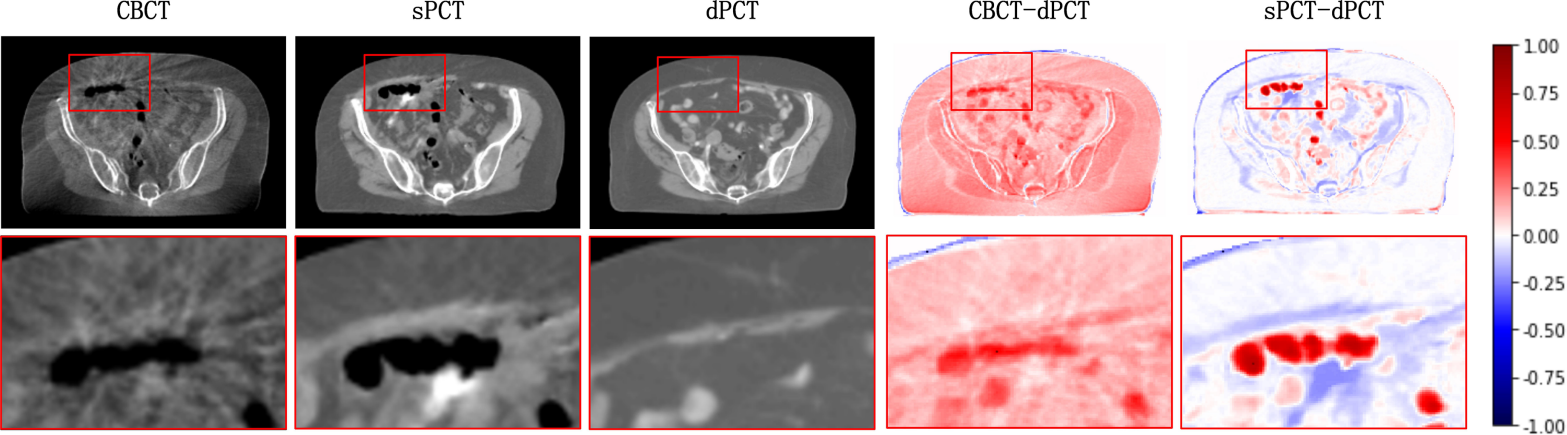
Artifact correction comparison. From left to right are CBCT, sPCT, dPCT, CBCT- dPCT, and sPCT - dPCT. The second row shows an enlarged version of the red rectangle of the image on the first row.

**Figure 6:**
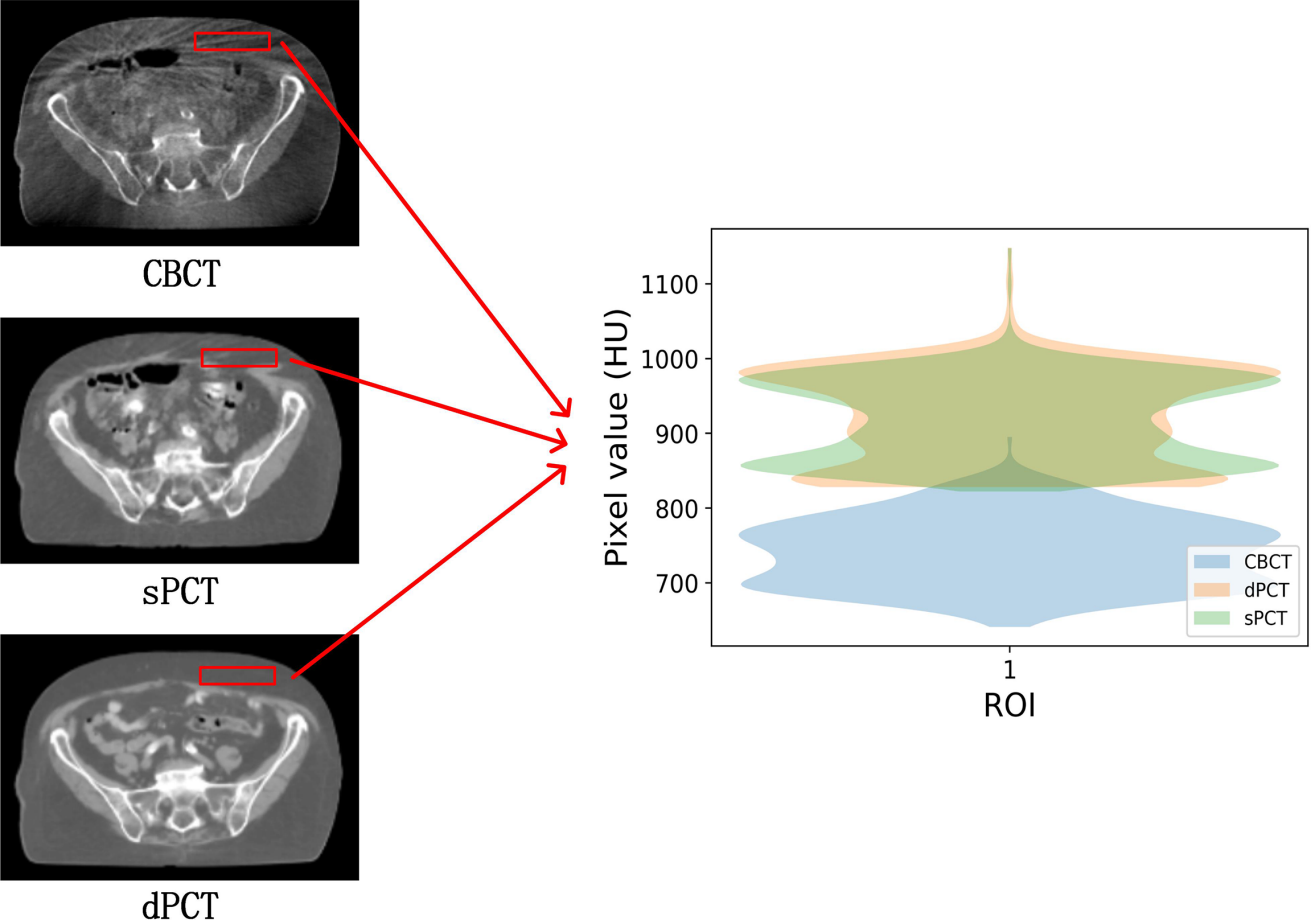
Comparison of ROI’s CT number distribution. The selected ROI is 70 × 20 pixels. The right part of the figure is the difference mapping of the CT number distribution between CBCT and dPCT, as well as sPCT and dPCT, respectively.

**Figure 4** shows the CBCT, sPCT, and dPCT images of two patients. We can see that sPCT can improve the image quality and spatial uniformity while keeping the image anatomical structure unchanged. On the whole, the image quality of sPCT is obviously better than that of CBCT. And the air space around the abdomen was observed to be significantly improved. Due to the error of registration, the anatomical structure between CBCT and dPCT is slightly different, whereas the anatomical structure of sPCT is keeping the same as that of CBCT, which indicates that sPCT images fully retain the anatomical information of CBCT images. **Figures 5** and **6** show the cases with large scattering artifacts in CBCT images. sPCT can not only suppress artifacts and restore the fine structure of images but also correct CT number of CBCT. It can be seen from **Figure 5** that the scattering artifacts in ROI and the whole image are well eliminated. The difference between sPCT and dPCT is less than the difference between dPCT and CBCT. **Figure 6** compares the CT number distribution of the selected ROI. It is also demonstrated that the difference of CT number distribution between sPCT and dPCT is less than that between CBCT and dPCT.

### Quantitative Evaluation of Image Quality

The quantitative results of all test cases are summarized in **Table 1**, which shows the difference between CBCT and dPCT, sPCT, and dPCT, respectively. Compared with the difference between CBCT and dPCT, the MAE, RMSE of the difference between sPCT and dPCT are only 14.6 ± 2.39 HU, 56.05 ± 13.05 HU, respectively, and PSNR is increased to 32.5 ± 1.87 db, which demonstrates that sPCT is more similar to dPCT. However, the increase of SSIM values is limited, because the structure of the dPCT obtained by registration is slightly different from that of the CBCT.

**Table 1:** Mean and standard deviation of CBCT - dPCT and sPCT - dPCT in all 9 test patients.

	Method	MAE (HU)	RMSE (HU)	PSNR (db)	SSIM
Patient 1	CBCT - dPCT	48.25	110.61	26.5	0.69
	sPCT - dPCT	16.49	54.25	31.38	0.82
Patient 2	CBCT - dPCT	48.75	100.51	26.75	0.73
	sPCT - dPCT	13.47	50.09	34.02	0.84
Patient 3	CBCT - dPCT	50.22	111.73	26.55	0.73
	sPCT - dPCT	16.24	52.23	32.25	0.83
Patient 4	CBCT - dPCT	48.23	104.14	26.46	0.68
	sPCT - dPCT	13.96	59.89	32.02	0.8
Patient 5	CBCT - dPCT	52.02	105.75	27.09	0.72
	sPCT - dPCT	15.49	57.19	32.4	0.84
Patient 6	CBCT - dPCT	52.85	102.34	26.76	0.74
	sPCT - dPCT	15.18	57.62	33.85	0.82
Patient 7	CBCT - dPCT	47.8	110.76	26.12	0.73
	sPCT - dPCT	14.34	52.64	32.56	0.81
Patient 8	CBCT - dPCT	51.18	106.42	26.58	0.73
	sPCT - dPCT	15.86	59.86	34.42	0.84
Patient 9	CBCT - dPCT	50.08	111.71	26.35	0.71
	sPCT - dPCT	13.6	58.99	30.89	0.84
Mean ± standard	CBCT - dPCT	49.96 ± 7.21	105.9 ± 11.52	26.82 ± 0.638	0.728 ± 0.36
	sPCT - dPCT	14.6 ± 2.39	56.05 ± 13.05	32.5 ± 1.87	0.825 ± 1.92

The SSIM is also used to evaluate between CBCT-dPCT, sPCT-dPCT, and sPCT-CBCT, respectively, as shown in **Table 2**. The SSIM of sPCT-CBCT is 0.882 ± 0.97, which is significantly higher than that of sPCT-dPCT, indicating that the structure of sPCT and CBCT was similar. It also proves that the generated sPCT can improve the image quality without changing the structure of CBCT, which has great clinical importance for ART radiotherapy.

**Table 2:** SSIM comparison of CBCT - dPCT, sPCT - dPCT, and sPCT - CBCT.

Evaluation	CBCT - dPCT	sPCT - dPCT	sPCT - CBCT
SSIM	0.728 ± 0.36	0.825 ± 1.92	0.882 ± 0.97

**Figure 7:**
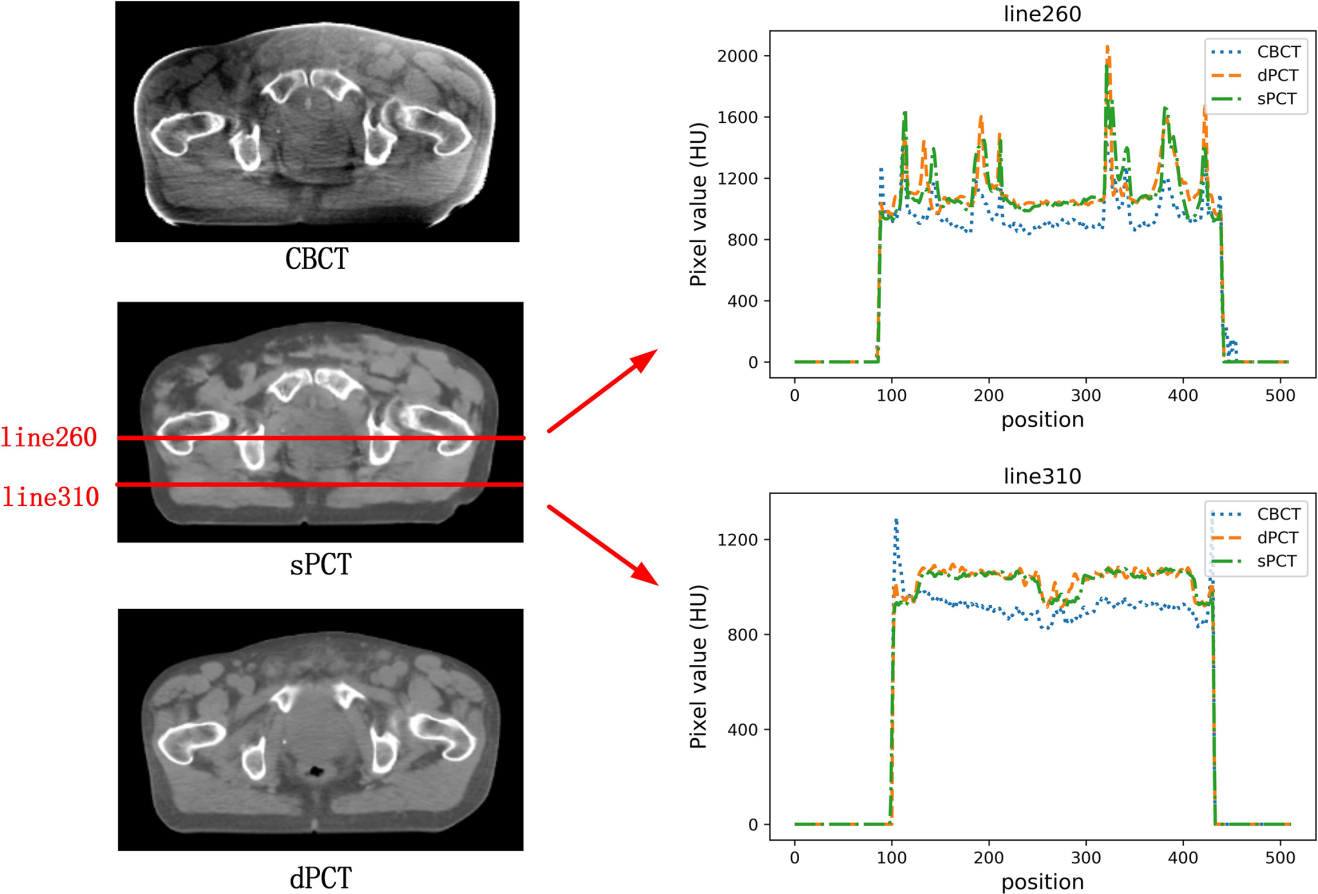
The left part is the axial view of CBCT, sPCT, and dPCT from top to bottom respectively. The right part is the distribution of CT number on two red lines (line 260 and line 310) on sPCT, CBCT, and dPCT respectively. Line 260 passed through bone and soft tissue, and line 310 only passed through soft tissue. The display window is (700, 1300) HU.

In the aspect of improvement of CT number, the overall CT number distribution of sPCT is close to that of dPCT. **Figure 7** shows the profiles of two red lines to evaluate the improvement of CT number. Line 260 passes through the soft tissue and bone area. It shows that the CT number of dPCT and sPCT are highly consistent, which are different from that of CBCT. In line 310, the contour only passes through the soft tissue area, and the CT number of CBCT is noisy, the results show that the CT number of the sPCT is not only corrected to the CT number of dPCT but also as smooth as the CT number of dPCT.

**Figure 8:**
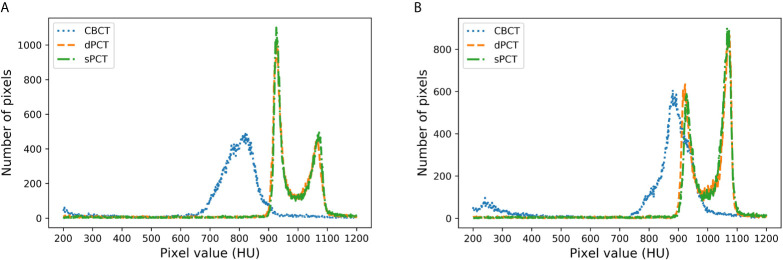
HU histograms of CBCT, sPCT, and dPCT respectively. The left and right images represent two patients **(A, B)**. For each patient, it displays the histogram of CBCT, dPCT, and sPCT images between 200HU and 1200HU.

**Figure 8** shows the HU histograms of CBCT, sPCT, and dPCT in two cases. Compared with dPCT and CBCT, the CT number difference between dPCT and sPCT is very small. Throughout the image, the CT number distribution of sPCT was similar to that of dPCT, indicating that the CT number of generated sPCT and dPCT are highly consistent.

**Figure 9:**
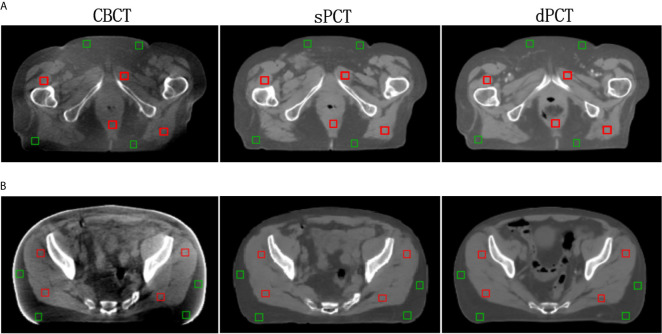
Distribution of the region of interests (ROIs). Two rows **(A, B)** represent the axial images of two patients respectively. The left, middle, and right images are CBCT, sPCT, and dPCT respectively. The green rectangle represents the fat ROIs, and the red rectangle represents the muscle ROIs. The corresponding ROI is placed in the same position on the CBCT, sPCT, and dPCT respectively. Each ROI contains 15 × 15 pixels. The display window is (700, 1300) HU.

**Figure 10:**
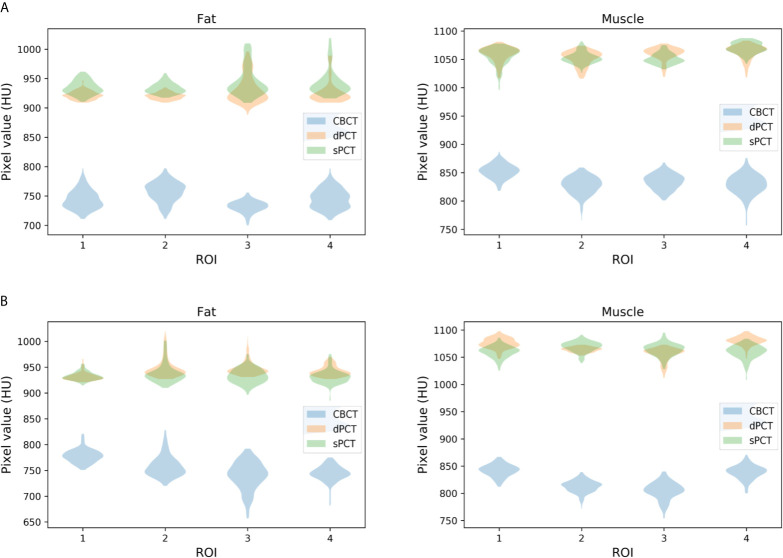
Distribution of CT number of CBCT, sPCT, and dPCT in muscle and fat ROIs. The first and second rows represent two patients **(A, B)**. The left and right columns represent the distribution of CT number in fat and muscle respectively. The number on the x-axis represents the number of ROIs in the same tissue, and the width represents the ratio of pixels with a specific HU.

Furthermore, four ROIs of fat and muscle tissue were selected to quantitatively evaluate the improvement of the CT number of each tissue of patients. The ROIs (15 × 15 pixels) were placed in the fat area of the selected CBCT slice, and the corresponding ROIs were placed in the same position on the corresponding slice of the sPCT and dPCT images. The same is for another evaluation ROIs (15 × 15 pixels) placed in the muscle area. The corresponding distribution of ROIs can be seen in **Figure 9**. The green and red rectangles represent the ROIs of adipose and muscle, respectively. **Figure 10** shows the comparison of CT number distribution of each ROI in CBCT, sPCT, and dPCT images, respectively. The width represents the ratio of pixels with a specific HU. The wider the width is, the larger the proportion of CT number is. It is not difficult to find that for all ROIs, the HU distribution of sPCT is closer to dPCT.

**Table 3** summarizes the mean, standard deviations and MAE of CT numbers for CBCT, sPCT, and dPCT. In the ROIs of fat tissue, the mean difference of CT numbers between CBCT and dPCT was 175.14 HU, the mean difference of CT numbers between sPCT and dPCT was only 1.65 HU, the mean MAE between CBCT and dPCT was 60.23 ± 7.3 HU, and the mean MAE between sPCT and dPCT was 12.94 ± 7.5 HU. In the ROIs of muscle tissue, the mean difference of CT numbers between CBCT and dPCT was 212.11 HU, and the mean difference of CT number between sPCT and dPCT was 11.07 HU. The mean MAE between CBCT and dPCT was 53.16 ± 9.1 HU, and the mean MAE between sPCT and dPCT was 13.03 ± 2.63 HU. It shows that whether in fat or muscle tissue, the CT number of sPCT is very close to dPCT than that of CBCT.

**Table 3:** Mean, standard deviation, and MAE of CT numbers of ROIs in CBCT, sPCT, and dPCT.

ROI	Evaluation	CBCT	sPCT	dPCT
Fat	Mean	749.53	926.32	924.67
	SD	82.35	34.36	43.92
	MAE	60.23 ± 7.3	12.94 ± 7.5	
Muscle	Mean	856.51	1057.55	1068.62
	SD	95.75	54.9	60.21
	MAE	53.16 ± 9.1	13.03 ± 2.63	

## Discussion

In this work, we applied the unsupervised deep learning network CycleGAN for CBCT scatter correction of the pelvis, which can be used for unpaired data. From **Figure 4**, it can be seen that the sPCT images obtained by this method has clear organ boundary, fewer scattering artifacts, and good uniformity. **Table 1** shows that the average MAE of CBCT and dPCT is 49.96 ± 7.21HU, and the average MAE of sPCT and dPCT is only 14.6 ± 2.39HU. The error is reduced by 35 HU after correction. The corresponding RMSE, PSNR, and SSIM also have obvious improvement. The MAE (14.6 ± 2.39HU) achieved in this study has a significant improvement compared with other studies in the introduction.

The proposed method does not force the organs in the sPCT images to be in the same position as in the dPCT images, but focuses much more on reducing the artifacts of CBCT tissue and improving the CT number accuracy of CBCT. From **Table 2**, it can be seen that the SSIM between CBCT and sPCT can reach 0.882 ± 0.97, which demonstrates that this method can improve the CT number accuracy of CBCT image while keeping the anatomical structure consistent.

The artifacts of CBCT have been effectively suppressed using the proposed method. It can be seen that sPCT has good spatial uniformity. Although only axial slices are used in the training process, the continuity of the structure and CT number is also kept in the coronal and sagittal planes as shown in **Figure 4**. To more clearly observe the effect of artifact suppression, we enlarged ROI with 60 × 50 pixels at the same position of CBCT and sPCT slices, and no obvious artifacts can be observed in sPCT images with better spatial uniformity (**Figure 5**).

**Figure 11:**
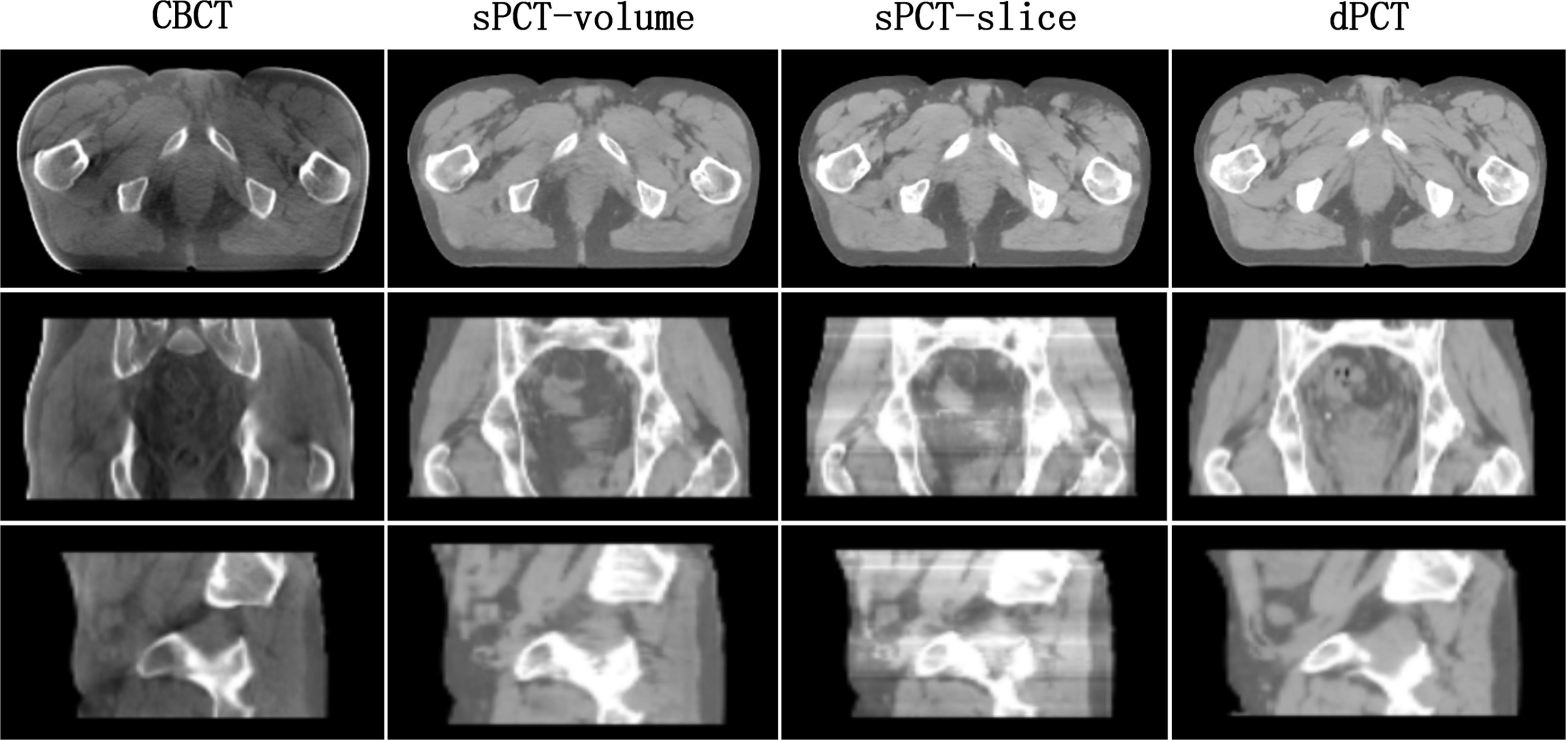
Effect of different normalization method on sPCT. Each column from left to right represents CBCT, sPCT (3D), sPCT (2D), and dPCT respectively. The top, middle, and bottom rows represent axial, coronal, and sagittal images respectively. Strip artifacts were found in the coronal and sagittal planes of 2D sPCT images. The display window is (700, 1300) HU.

In the process of CycleGAN network training, different normalization methods were used for different data preprocessing: (a) normalize the 3D volume data of CBCT and PCT (see equation 10); (b) normalize the 2D slice data of CBCT and PCT (see equation 11). The visual comparison of different normalized sPCT images is shown in **Figure 11**. The result using the first normalization method (a) does not have the strip shape artifact with good spatial uniformity, whereas there are many strip shape artifacts in the sagittal and coronal planes of 3D sPCT when using the second normalization method (b), which significantly affect the image quality. The reason is that *Data_slice–max_* – *Data_slice–min_* in each slice is different from that in other slices for the second normalization method, which results in stripe artifacts when generating 3D data.

(10) $$\text{Data}=\frac{Data_{volume}-Data_{volume-min}}{Data_{volume-max}-Data_{volume-min}}$$

(11) $$\text{Data}=\frac{Data_{slice}-Data_{slice-min}}{Data_{slice-max}-Data_{slice-min}}$$

Generally, the following three points are essential when applying the deep learning model in the clinic. (a) Accuracy: the sPCT images obtained by this method is close to the actual dPCT images, especially in the aspect of artifact suppression and CT number accuracy, which outperforms other traditional methods; (b) computational speed: the modern clinical treatment requires higher computing speed of medical images. Once the training is completed, it only takes a few seconds in the clinical treatment, which is much faster than traditional methods. (c) Generalization: the trained network needs to meet the requirements that the data acquired on different devices can still work. However, the trained model in this study can only work on data of the pelvic area under the same device, and it does not have universality to be utilized under other conditions. The limitation will be improved in the near future.

In our CycleGAN network, the appearance of the target is enforced by adversarial loss, while the content is retained by cycle consistency loss. Cycle consistency loss assumes that the relationship between two domains (CBCT and PCT) is bijective, which is usually too strict. For some organs or tissues with artifacts and inaccurate CT numbers in sPCT, such a requirement is difficult to satisfy. For example, the surrounding tissues with artifacts in CBCT ROI in **Figure 6** need to be changed on sPCT, to improve the spatial uniformity of images. To tackle this issue, Zhu (53) proposed a multi-layer patch-based method to maximize the mutual information between the corresponding input and output patches to maintain the correspondence in the content, which achieved good results in the zebra horse experiment. The method mentioned above is called CUT. Surprisingly, this method can even be extended to complete training with only one data (each “domain” is just an image).

Based on this, there are two works worth studying in the future (1): to compare and evaluate the application of CycleGAN and CUT network in medical data set, and integrate the advantages of these two methods (2); CUT uses the multi-layer patch-based method to select different patches from the input image itself for training, so it can train on a single image, which is incomparable to CycleGAN, as medical data are challenging to obtain, we will use one data (one CBCT image and one PCT image) to train and evaluate the CUT network (3); We applied 49 CBCT images from different patients for model training. The results are found to be acceptable. In the future, effectively enlarging the training images is critically important for improving the accuracy of artifact correction and avoiding overfitting.

## Conclusions

In this study, the CycleGAN model was used to generate high-quality synthetic PCT images from CBCT images. The feasibility of synthesized PCT imaging in clinical radiotherapy for pelvic patients was preliminarily verified and evaluated, and satisfactory and clinically acceptable results were obtained. The proposed method in this paper illustrates the potential of clinical ART application in the near future. In addition, the work described in this paper can be further extended to the application in other parts of the human body.

## Data Availability Statement

Publicly available datasets were analyzed in this study. These data can be found here: https://wiki.cancerimagingarchive.net/display/Public/Pelvic+Reference+Data.

## Ethics Statement

Written informed consent was obtained from the individual(s) for the publication of any potentially identifiable images or data included in this article.

## Author Contributions

CZ wrote the manuscript and developed most of the algorithm code. GD was responsible for the analysis of patient data and the revision of some manuscripts before submission. XL proposed the original notion and supervised the research, and LD developed part of the algorithm code. LS discussed the algorithm details and revised the revision. YZ and XYZ revised the manuscript. XRZ and XZ were responsible for proofreading. YX helped allocate the writing time for the author’s clinical work. All authors contributed to the article and approved the submitted version.

## Funding

This work is supported in part by grants from the National Key Research and Develop Program of China (2016YFC0105102), the Leading Talent of Special Support Project in Guangdong (2016TX03R139), Shenzhen matching project (GJHS20170314155751703), the Science Foundation of Guangdong (2017B020229002, 2015B020233011) and CAS Key Laboratory of Health Informatics, Shenzhen Institutes of Advanced Technology, and the National Natural Science Foundation of China (61871374, 81871433, 61901463, 90209030, C03050309), Shenzhen Science and Technology Program of China grant (JCYJ20170818160306270), Fundamental Research Program of Shenzhen (JCYJ20170413162458312), the Special research project slot son of basic research of the Ministry of Science and Technology (No. 22001CCA00700), the Guangdong Provincial Administration of Traditional Chinese Medicine (20202159).

## Conflict of Interest

The authors declare that the research was conducted in the absence of any commercial or financial relationships that could be construed as a potential conflict of interest.
